# Factors associated with the dietary total antioxidant capacity of pregnant Brazilian women

**DOI:** 10.1590/1980-549720250002

**Published:** 2025-02-10

**Authors:** Roberta Rejane Santos de Carvalho, Poliana Cristina de Almeida Fonseca Viola, Sandra Patrícia Crispim, Ana Karina Teixeira da Cunha França, Anderson Marliere Navarro, Bruno Feres de Souza, Franciane Rocha de Faria, Naiara Sperandio, Nathalia Pizato, Mariana de Souza Macedo, Renata Junqueira Pereira, Sylvia do Carmo Castro Franceschini, Carolina Abreu de Carvalho, *Multicenter Study on Iodine Deficiency (Estudo Multicêntrico de Deficiência de Iodo - EMDI Brasil), Anderson Marliere Navarro, Carolina Abreu de Carvalho, Danielle Góes da Silva, Franciane Rocha de Faria, Naiara Sperandio, Jorge Gustavo Velásquez Meléndez, Míriam do Carmo Rodrigues Barbosa, Nathalia Pizato, Mariana de Souza Macedo, Renata Junqueira Pereira, Sandra Patrícia Crispim, Silvia Eloiza Priore, Sylvia do Carmo Castro Franceschini

**Affiliations:** I Universidade Federal do Maranhão, Graduate Program in Public Health - São Luís (MA), Brazil.; II Universidade Federal do Piauí, Department of Nutrition, Ministro Petrônio Portella Campus - Teresina (PI), Brazil.; III Universidade Federal do Paraná, Graduate Program in Food and Nutrition - Curitiba (PR), Brazil.; IV Universidade de São Paulo, School of Medicine of Ribeirão Preto, Department of Health Sciences - Ribeirão Preto (SP), Brazil.; V Universidade Federal de Rondonópolis, School of Health Sciences - Rondonópolis (MT), Brazil.; VI Universidade Federal do Rio de Janeiro - Macaé (RJ), Brazil.; VII Universidade Federal de Brasília, Department of Nutrition - Brasília (DF), Brazil.; VIII Universidade Federal dos Vales do Jequitinhonha e Mucuri, Graduate Program in Nutrition Sciences - Diamantina (MG), Brazil.; IX Universidade Federal do Tocantins, Nutrition Outpatient Clinic - Palmas (TO), Brazil.; X Universidade Federal de Viçosa, Graduate Program in Nutrition Science - Viçosa (MG), Brazil.; i Universidade de São Paulo – Ribeirão Preto (SP), Brazil.; ii Universidade Federal do Maranhão – São Luís (MA), Brazil; iii Universidade Federal de Sergipe – Aracaju (SE),Brazil.; iv Universidade Federal de Rondonópolis – Rondonópolis (MT), Brazil; v Universidade Federal do Rio de Janeiro – Macaé (RJ), Brazil.; vi Universidade Federal de Minas Gerais – Belo Horizonte (MG), Brazil.; vii Universidade Federal do Espírito Santo – Vitória (ES), Brazil; viii Universidade de Brasília – Brasília (DF), Brazil; ix Universidade Federal dos Vales do Jequitinhonha e Mucuri – Diamantina (MG), Brazil; x Universidade Federal do Tocantins – Palmas (TO), Brazil; xi Universidade Federal do Paraná – Curitiba(PR), Brazil.; xii Universidade Federal de Viçosa – Viçosa (MG), Brazil; xii Universidade Federal de Viçosa – Viçosa (MG), Brazil

**Keywords:** Antioxidants, Pregnancy, Oxidative stress, Food consumption, Sociodemographic factors

## Abstract

**Objective:**

To investigate the sociodemographic, maternal, and gestational factors associated with the dietary total antioxidant capacity in pregnant Brazilian women.

**Methods:**

A cross-sectional study with 2,232 pregnant women aged 18 years old or older, in the first, second, and third trimesters of pregnancy, from eleven cities in the five Brazilian regions. A semi-structured questionnaire was applied to assess socioeconomic, demographic, and health data, and a 24-hour dietary recall (R24h) was used to assess food consumption and analyze the dietary total antioxidant capacity (DTAC), estimated using the ferric reducing antioxidant power (FRAP) method.

**Results:**

The median of DTAC was 5.32 mmol/day. Aracaju, Sergipe (SE) had the highest median of DTAC (6.44 mmol/day) and Palmas, Tocantins (TO) had the lowest (4.71 mmol/day). Pregnant women aged 20 to 34 years (OR 1.86; 95%CI 1.26-2.76), 35 years old or older (OR 3.68; 95%CI 2.21-6.14) and who were in the second trimester of pregnancy (OR 1.50; 95%CI 1.11-2.01) were more likely to be above the median DTAC. While pregnant women with higher education had a 67% lower chance of being above the median DTAC (OR 0.67; 95%CI 0.48-0.92).

**Conclusion:**

The study demonstrated that there are differences in antioxidant consumption in different cities in Brazil and that associated factors such as age, education, and gestational trimester can impact the intake of foods rich in antioxidants. The profile found draws attention to the importance of an adequate diet rich in antioxidants during prenatal care.

## INTRODUCTION

Maternal nutrition is a key determinant of the fetus's proper development and growth, as this stage of life is characterized by increased energy and nutritional requirements^1,2^.

These needs arise from an increased basal metabolic rate, elevated cardiac output, heightened oxygen consumption, and the formation of free radicals, which characterize pregnancy as a state of high oxidative stress^3^. Consequently, a diet rich in nutrients with antioxidant properties, primarily sourced from fruits and vegetables, is recommended during pregnancy. Such a diet can help prevent maternal and fetal complications, including pre-eclampsia, diabetes, premature birth, spontaneous abortion, intrauterine growth restriction, and other related disorders^4-7^.

Evidence suggests that in Brazil, the dietary intake of pregnant women is often inadequate, marked by low consumption of fruits and vegetables^8^, alongside excessive intake of high-calorie foods with low levels of essential nutrients^9,10^.

The Dietary Total Antioxidant Capacity (DTAC) serves as a measure of dietary intake and a marker of diet quality, accounting for all antioxidants present in an individual's diet and their synergistic interactions^11-13^. Studies investigating DTAC in pregnant women are limited both in Brazil and globally, and no specific recommendations for antioxidant intake during pregnancy currently exist.

To date, only two studies have been identified that assessed DTAC in pregnant women, both conducted with the same sample in Ribeirão Preto, São Paulo (SP), Brazil^4,14^. Sartorelli et al.^4^ found that pregnant women with higher DTAC levels had a reduced likelihood of experiencing preterm birth and delivering newborns with low birth weight.

Food consumption is shaped by regional and socioeconomic differences, and Brazil's vast territorial extent contributes to significant social disparities. These disparities affect both access to quality food and dietary choices^15^. Although no studies have specifically evaluated DTAC across different regions of the country, research examining other aspects of dietary intake has identified regional variations^16,17^. For instance, Pereira et al.^16^ analyzed the Dietary Inflammatory Index (DII) of the Brazilian population and observed that residents of the Northeast and South regions had more pro-inflammatory diets, while those in the Central-West region exhibited the lowest DII. It is plausible that an analysis of DTAC could also reveal regional differences, which would help inform public policies tailored to regions at greater risk. Accordingly, this study aimed to investigate the sociodemographic, maternal, and gestational factors associated with the DTAC in Brazilian pregnant women.

## METHODS

This cross-sectional study utilized data from the Multicenter Study on Iodine Deficiency (*Estudo Multicêntrico de Deficiência de Iodo* - EMDI), conducted in cities across Brazil's five regions: Viçosa (Minas Gerais - MG), Belo Horizonte (MG), Vitória (Espírito Santo - ES), Macaé (Rio de Janeiro - RJ), Ribeirão Preto (SP), Pinhais (Paraná - PR), Brasília (Distrito Federal - DF), Rondonópolis (Mato Grosso - MT), São Luís (Maranhão - MA), Aracaju (Sergipe - SE), and Palmas (Tocantins - TO). The study evaluated pregnant women over 18 years of age, classified as normal-risk, who were receiving prenatal care through the Brazilian Unified Health System (*Sistema Único de Saúde* - SUS) across all trimesters of pregnancy. Pregnant women with a history of thyroid disease and/or surgery or a diagnosis of hypothyroidism were excluded from the study.

The EMDI sample size was determined using a minimum estimable proportion of 8%, with a margin of error of 50% (ranging from 4% to 12%) and a 95% confidence interval, resulting in a simple random sample of 177 pregnant women. A design effect of 1.5 was incorporated into the calculation to account for the complexity of the sample, as it was drawn from Basic Health Units (*Unidades Básica de Saúde* - UBS) in each municipality studied. Consequently, the minimum sample size was increased to 266 pregnant women at each collection center.

Participant selection was conducted in two stages: in the first stage, UBS in each city were randomly selected; in the second stage, pregnant women were chosen from a list of women monitored monthly throughout the study period, which served as the basis for the draw and recruitment. In some units, where it was not possible to obtain the list of pregnant women, participants were selected randomly from the list of women scheduled for routine prenatal consultations, based on the demand for care on the day the research team visited the UBS.

Data were collected from January 2019 to March 2021 at UBSs in eleven cities by trained interviewers. Pregnant women were interviewed either while waiting for their routine prenatal appointment or after it. In cases where the pregnant woman was unable to complete the interview at the UBS, the interview was rescheduled to take place at her home.

A questionnaire administered via tablets, using the RedCap^®^ application, was employed to collect socioeconomic, demographic, and health data. This included information on regions (North, Northeast, Central-West, Southeast, and South), age range (18 to 19 years, 20 to 34 years, 35 years old or older), family income in reais (categorized into tertiles), skin color (white and non-white), living with a partner (no; no, but has lived with a partner; yes), education level (0 to 8 years, 9 to 11 years, 12 or more), head of household (partner, self, and others), place of residence (rural and urban), gestational trimester (first, second, and third), pre-gestational BMI in kg/m^2^ (underweight, normal weight, and overweight), and gestational BMI (underweight, adequate, overweight). The weight and height used to calculate pre-pregnancy BMI were self-reported by the pregnant women and were also checked against the information on pregnant woman's card, when available. In cases where discrepancies were found between self-reported data and the information on the card, priority was given to the latter, as it was provided by the health professional.

The cutoff points used to classify pre-gestational nutritional status were based on the World Health Organization (WHO) criteria: <18.5 kg/m^2^ (underweight), 18.5 to 24.9 kg/m^2^ (normal weight), 25.0 to 29.9 kg/m² (overweight), and ≥30 kg/m² (obesity)^18^. In this study, the excess weight category included women classified as overweight or obese.

Gestational nutritional status was determined by calculating the BMI using the pregnant woman's current weight, which was then classified according to the BMI curve for gestational age^19^.

The estimation of pregnant women's food consumption was conducted using a 24-hour recall (R24h) and applying the “multiple-pass method”^20^. The R24h included details about the timing of each meal, whether the food consumed was homemade or industrialized, the brand, type or flavor, preparation method, recipes for each dish, and the quantities consumed.

The Photographic Manual of Food Quantification was used to quantify the portions of each food item or preparation. This manual contains 96 photographs of food portions, typical Brazilian dishes, and various methods of food quantification^21^.

The 24-hour recalls from all research centers were sent to the Food Exposure Research Group (*Grupo de Pesquisa em Exposição Alimentar* - GUPEA) at Universidade Federal do Paraná, where they were entered and analyzed using the GloboDiet software. Nutritional components were identified using both the Brazilian Food Composition Table (*Tabela Brasileira de Composição de Alimentos* - TBCA) and the Food Iodine Composition Table (*Tabela de Composição de Iodo em Alimentos* - TCIA)^22,23^.

The TAC of each food item was calculated by multiplying the quantity of the food or drink reported in the R24h by its Ferric Reducing Antioxidant Power (FRAP) value, then dividing the result by 100. Once the TAC values for each food and drink were determined, they were summed to calculate the DTAC for each pregnant woman on the reported day.

The DTAC was estimated using databases developed by Carlsen et al.^24^ and Halvorsen et al.^25^, as well as a Brazilian table created by Rufino et al.^26^, which focuses on analyzing the antioxidants of 18 native, non-traditional, and fresh fruits. These databases describe the TAC of various foods and beverages, using the FRAP method. FRAP values for fruits were primarily sourced from the Brazilian table^26^. For foods with multiple FRAP values in the databases, an average was calculated. If a specific food was not listed in the tables, the FRAP value of a food from the same botanical group or with similar nutritional properties was used.

A descriptive analysis was conducted for sociodemographic variables, gestational trimester, and pre-gestational and gestational nutritional status. Categorical variables were presented as absolute and relative frequencies, while continuous variables were described using measures of central tendency and dispersion. Normality of the variable distributions was assessed through histograms, boxplot graphics, and the Shapiro-Wilk test.

DTAC values were adjusted for energy intake and categorized into tertiles for analysis, as no established cutoff points for DTAC classification currently exist. Nutrient intake among the pregnant women was also adjusted for energy using the residual method. Comparisons of nutrient amounts across DTAC tertiles were conducted using the Kruskal-Wallis test. To examine sociodemographic differences between DTAC tertiles, the chi-square test was applied for categorical variables, while the Kruskal-Wallis test was used for continuous variables.

The comparison of coffee consumption medians across education groups, as well as DTAC medians between regions and cities, was conducted using the Kruskal-Wallis test. The Dunn post-hoc test was applied to identify differences among education groups, regions, and cities.

Factors associated with DTAC above the median were analyzed using Odds Ratios (OR) calculated through bivariate and multivariate logistic regression models. The OR values from the logistic regression assessed the factors linked to DTAC above the median, as detailed in Table 1. Explanatory variables included in the multivariate model were those with a p-value <0.10 in the bivariate analysis, specifically: age, education, living with a partner, gestational trimester, and pre-gestational BMI. Data analysis was performed using RStudio^®^ software, version 4.2.1. A significance level of 5% was applied to all analyses, except for the bivariate models.

**Table 1:** Factors associated with dietary total antioxidant capacity above the median (>5.32 mmol/day) among pregnant women participating in the Multicenter Study on Iodine Deficiency, by regions of Brazil. Brazil, 2019.

		Dietary Total Antioxidant Capacity *					
		Unadjusted model			Adjusted model †		
		OR	95%CI	p-value	OR	95%CI	p-value
Regions							
	Central-West	1	–				
	North	0.74	0.40-1.36	0.343			
	Northeast	1.17	0.84-1.62	0.337			
	Southeast	1.25	0.92-1.68	0.143			
	South	1.02	0.69-1.50	0.903			
	*Per capita* income	1.00	0.99-1.00	0.218			
Age (years)							
	18 to 19	1	–		1	–	
	20 to 34	1.50	1.05-2.15	**0.024**	1.86	1.26-2.76	**0,001**
	35 or more	2.75	1.75-4.33	**<0.001**	3.68	2.21-6.14	**<0,001**
Skin color							
	White	1	–				
	Non-White	1.02	0.80-1.31	0.825			
Lives with a partner							
	No	1	–		1	–	
	No, but has lived	1.58	0.98-2.54	**0.056**	1.28	0.77-2.12	0,325
	Yes	1.01	0.74-1.39	0.921	0.85	0.61-1.18	0,342
Education (years)							
	0 to 8	1	–		1	–	
	9 to 11	0.78	0.55-1.10	0.163	0.79	0.54-1.15	0,226
	12 or	0.69	0.51-0.93	**0.015**	0.67	0.48-0.92	**0,014**
Head of the household							
	Partner	1	–				
	Herself	1.20	0.93-1.54	0.152			
	Others	0.96	0.74-1.25	0.808			
Place of residence							
	Rural	1	–				
	Urban	1.34	0.86-2.08	0.192			
Gestational trimester							
	First	1	–		1	–	
	Second	1.51	1.15-1.98	**0.003**	1.50	1.11-2.01	**0,006**
	Third	1.30	0.99-1.71	0.050	1.28	0.96-1.71	0,087
Pre-pregnancy BMI							
	Normal weight	1	–		1	–	
	Underweight	0.60	0.37-0.99	**0.045**	0.68	0.41-1.13	0,144
	Overweight	0.92	0.74-1.15	0.515	0.86	0.69-1.09	0,231
Current gestational BMI							
	Normal weight	1	–				
	Underweight	0.77	0.54-1.09	0.146			
	Overweight	0.91	0.71-1.16	0.450			

* DTAC by energy using the residual method. Median DTAC 5.32 mmol/day;

† Logistic regression model adjusted for age (0-19/20-34/35 years old or older), living with a partner (no; no, but has lived; yes), education (0-8 years/9-11 years/12 or more years), gestational trimester (first, second, and third), and pre-gestational body mass index (kg/m^2^) (underweight, normal weight, and overweight).

DTAC: Dietary total antioxidant capacity; OR: odds ratio; CI: confidence interval; BMI: body mass index.

Bold values with p<0.05.

This project was approved by the Human Research Ethics Committee of Universidade Federal de Viçosa, the coordinating institution for the multicenter study (CAAE: 80172617.0.1001.5153). Additionally, all participating centers submitted the project to their respective local ethics committees (CEP) and received approval.

## RESULTS

A total of 2,376 pregnant women participated in the EMDI, with 2,247 completing the R24h. Among these, 15 were excluded due to missing sociodemographic information, resulting in 2,232 participants included in the present study (response rate = 93.9%). Of these, 43% resided in the Southeast region, the majority (78%) were aged 20-34 years, with a median per capita income of R$600.00. Additionally, 76.6% identified as non-white, 78.8% lived with a partner, and 62.2% had 12 or more years of education. Regarding pregnancy characteristics, 39.8% were in the third trimester, and 47.2% were classified as overweight (Table 2).

**Table 2:** Socioeconomic and health characteristics by tertile of dietary total antioxidant capacity (DTAC) adjusted for energy in pregnant women participants of the Multicenter Iodine Deficiency Study (*Estudo Multicêntrico de Deficiência de Iodo* - EMDI Brazil), according to regions of Brazil. Brazil, 2019.

		Total	T1 (lowest; n=744)	T2 (n=744)	T3 (highest; n=744)	p-value
		n (%)	n (%)	n (%)	n (%)	
Regions						
	North	89 (4.0)	32 (36.0)	36 (40.4)	21 (23.6)	0.154*
	Northeast	553 (24.8)	189 (34.2)	169 (30.6)	195 (35.3)	
	Central-West	357 (16.0)	125 (35.0)	122 (34.2)	110 (30.8)	
	Southeast	960 (43.0)	297 (30.9)	336 (35.0)	327 (34.1)	
	South	273 (12.2)	101 (37.0)	81 (29.7)	91 (33.3)	
Age range (years)						
	18 to 19	219 (9.8)	86 (39.3)	78 (35.6)	55 (25.1)	**0.001** *
	20 to 34	1,742 (78.0)	590 (33.9)	583 (33.5)	569 (32.7)	
	35 or more	271 (12.2)	68 (25.1)	83 (30.6)	120 (44.3)	
Family income (tertiles)						
	Median	600	566.66	570.83	600	0.805†
Skin color						
	White	491 (23.4)	173 (35.2)	150 (30.5)	169 (34.4)	0.386*
	Non-White	1,607 (76.6)	532 (33.1)	544 (33.9)	531(33.0)	
Lives with a partner						
	No	277 (13.3)	92 (33.3)	98 (35.4)	87 (31.4)	0.159*
	No, but has lived	165 (7.9)	46 (27.9)	50 (30.3)	69 (41.8)	
	Yes	1,645 (78.8)	561 (34.1)	545 (33.1)	539 (32.8)	
Education (years)						
	0 to 8	344 (16.5)	95 (27.6)	124 (36.0)	125 (36.3)	0.103*
	9 to 11	445 (21.3)	150 (33.7)	141 (31.7)	154 (34.6)	
	12 or more	1,299 (62.2)	458 (35.3)	424 (32.6)	417 (32.1)	
Head of the household						
	Partner	1,020 (48.6)	353 (34.6)	332 (32.5)	335 (32.8)	0.194*
	Herself	560 (26.7)	180 (32.1)	175 (31.3)	205 (36.6)	
	Others	518 (24.7)	172 (33.2)	188 (36.3)	158 (30.5)	
Place of residence						
	Rural	121 (5.8)	48 (39.7)	36 (29.8)	37 (30.6)	0.275*
	Urban	1,975 (94.2)	644 (32.6)	665 (33.7)	666 (33.7)	
Gestational trimester						
	First	505 (22.7)	189 (37.4)	171 (33.9)	145 (28.7)	0.053*
	Second	836 (37.5)	255 (30.5)	285 (34.1)	296 (35.4)	
	Third	887 (39.8)	299 (33.7)	288 (32.5)	300 (33.8)	
Pre-pregnancy BMI (kg/m^2^)						
	Underweight	113 (5.6)	47 (41.6)	36 (31.9)	30 (26.5)	0.112*
	Normal weight	946 (47.2)	300 (31.7)	332 (35.1)	314 (33.2)	
	Overweight	946 (47.2)	330 (34.9)	295 (31.2)	321 (33.9)	
Gestational BMI						
	Underweight	276 (15.2)	94 (34.1)	104 (37.7)	78 (28.3)	0.235*
	Normal weight	683 (37.6)	223 (32.7)	220 (32.2)	240 (35.1)	
	Overweight	859 (47.2)	296 (34.5)	273 (31.8)	290 (33.8)	

* Pearson's Chi-squared test;

† Kruskal-Wallis rank sum test. Bold values indicate p<0.05.

**Figure 1:**
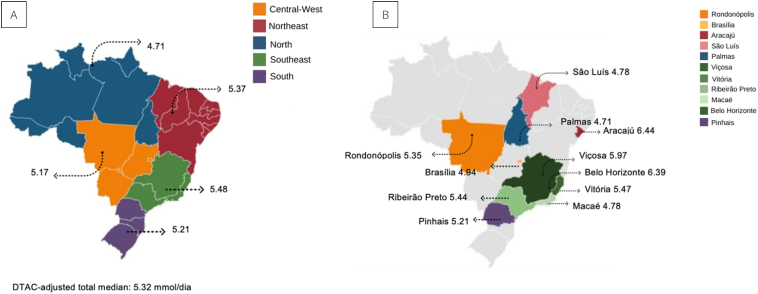
Median of dietary total antioxidant capacity adjusted for pregnant women in the Iodine Deficiency Multicenter Study. A) Median dietary total antioxidant capacity in mmol/day adjusted for energy of pregnant women, according to the region of Brazil, and B) Median dietary total antioxidant capacity in mmol/day adjusted for energy of pregnant women, according to the city. Brazil, 2019.

The median DTAC was 5.32 mmol/day. Among the regions, the Southeast recorded the highest median DTAC (5.48 mmol/day), while the North had the lowest (4.71 mmol/day) (Figure 1). At the city level, Aracaju (SE) had the highest median DTAC (6.44 mmol/day), whereas Palmas (TO) reported the lowest median (4.71 mmol/day).

DTAC: Dietary total antioxidant capacity

A significant difference in DTAC medians was observed between the cities (p<0.001). The median DTAC in Palmas was lower than in Aracaju (SE) and Belo Horizonte (MG). However, no significant difference was found in the DTAC medians among the regions (Figure 1).

Pregnant women aged 35 years old or older were more likely to be in the highest DTAC tertile (p=0.001), as shown in Table 2. Additionally, those with higher consumption of carbohydrates, fiber, iron, magnesium, potassium, copper, retinol, vitamin D, vitamin E, thiamine, vitamin B2, vitamin B6, folate, and iodine were also more likely to fall into the highest DTAC tertile (Table 3).

**Table 3:** Dietary characteristics according to the tertiles of the estimated dietary total antioxidant capacity adjusted for energy of pregnant women participating in the Multicenter Iodine Deficiency Study, by regions of Brazil. Brazil, 2019.

Tertiles of adjusted DTAC				
Nutrients	T1 (Lowest)	T2	T3 (Highest)	p-value*
Carbohydrates (g)	230.46	240.25	251.67	**<0.001**
Protein (g)	77.55	73.64	72.78	**0.001**
Lipids (g)	68.41	65.83	62.65	**<0.001**
Saturated fatty acids (g)	23.02	21.30	20.19	**<0.001**
Monounsaturated fatty acids (g)	20.81	19.59	18.64	**<0.001**
Polyunsaturated fatty acids (g)	15.54	15.74	15.32	0.269
Trans fatty acids (g)	1.52	1.44	1.37	**0.001**
Cholesterol (mg)	266.98	241.74	234.10	**<0.001**
Fiber (g)	14.36	18.58	20.68	**<0.001**
Calcium (mg)	448.06	467.28	461.26	0.435
Iron (mg)	10.16	10.70	11.16	**<0.001**
Sodium (mg)	2,326.23	2,293.26	2,252.85	0.214
Magnesium (mg)	211.47	239.85	262.85	**<0.001**
Phosphorus (mg)	992.18	1,003.61	1,010.64	0.336
Potassium (mg)	1,855.81	2,133.86	2,418.02	**<0.001**
Manganese (mg)	3.40	4.08	3.84	**<0.001**
Zinc (mg)	10.52	10.49	10.11	0.572
Copper (mg)	1.04	1.18	1.23	**<0.001**
Selenium (mcg)	36.51	36.58	35.90	0.328
Vitamin A (mcg)	333.99	372.16	406.15	**<0.001**
Vitamin D (mcg)	2.02	2.26	2.76	**<0.001**
Vitamin E (mcg)	5.25	5.68	5.89	**<0.001**
Thiamine (mg)	0.91	0.93	1.03	**<0.001**
B2 (mg)	0.99	1.15	1.31	**<0.001**
B3 (mg)	14.83	14.23	14.33	0.387
B6 (mg)	0.59	0.67	0.71	**<0.001**
B12 (mcg)	4.00	3.62	3.20	**<0.001**
Vitamin C (mg)	57.49	72.36	71.28	**<0.001**
Folate (eq)	293.10	351.56	409.17	**<0.001**
Iodine (mcg)	115.83	121.36	124.15	**0.002**

* Kruskal-Wallis rank sum test.

DTAC: Dietary total antioxidant capacity. Bold values indicate p<0.05.

The five foods with the highest mean DTAC contribution per center are detailed in Supplementary Table 1, while the foods with the highest median DTAC contribution by education categories and region are presented in Table 4.

**Table 4:** Foods with the highest median contributions to the Dietary Total Antioxidant Capacity in pregnant women participating in the Multicenter Iodine Deficiency Study, according to education level and region. Brazil, 2019.

Education		Median daily consumption (mmol/day)
0 to 8 years		
	Coffee	1.86
	Beans	0.93
	Rice	0.14
	White onion	0.02
	Bay leaves	0.00
9 to 11 years		
	Beans	0.97
	Coffee	0.93
	Rice	0.14
	White onion	0.02
	Bay leaves	0.00
12 years or more		
	Coffee	1.04
	Beans	0.85
	Rice	0.13
	White onion	0.01
	Bay leaves	0.00
	Region	Median daily consumption (mmol/day)*
	Beans	0.97
	Coffee	0.52
	Rice	0.14
Northeast		
	Coffee	1.8
	Beans	0.42
	Rice	0.13
Southeast		
	Beans	0.97
	Coffee	0.76
	Rice	0.14
Central-West		
	Beans	0.97
	Coffee	0.78
	Rice	0.14
South		
	Coffee	0.76
	Beans	0.39
	Rice	0.13

* the medians of the three foods that contributed the most to the Dietary Total Antioxidant Capacity by region were included, as the median values of the 4^th^ and 5^th^ foods were very small, appearing as zero due to rounding.

According to Supplementary Table 1, the average coffee contribution to DTAC in Aracaju was nearly double compared to some other centers.

There was a difference in median coffee consumption between education groups (p=0.009). The median coffee consumption of pregnant women with 0 to 8 years of education was higher than that of pregnant women with 9 to 11 years of education and those with 12 years or more of education.

In a positive gradient, adult pregnant women were more likely to have an antioxidant intake above the median DTAC, with those aged 20 to 34 years being 1.86 times more likely (OR 1.86; 95%CI 1.26-2.76) and those aged 35 years old or older being 3.68 times more likely (OR 3.68; 95%CI 2.21-6.14). Pregnant women with higher education (OR 0.67; 95%CI 0.48-0.92) were 67% less likely to be above the median DTAC compared to pregnant women with lower education (Table 1). Additionally, those in the second trimester were 1.5 times more likely to be above the median DTAC (OR 1.5; 95%CI 1.11-2.01) compared to those in the first trimester (Table 1).

## DISCUSSION

This study evaluated the factors associated with DTAC in Brazilian pregnant women using the public health system and found that the median DTAC was 5.32 mmol/day. Pregnant women aged 35 years old or older had a greater likelihood of having antioxidant intake above the median DTAC. Additionally, pregnant women in the second trimester were more likely to exceed the median DTAC. However, it was observed that those with higher education were less likely to have DTAC values above the median. The Southeast region had the highest median DTAC.

Among the Brazilian cities evaluated in this study, Aracaju (SE) had the highest DTAC (6.44 mmol/day), which can be attributed to the higher consumption of coffee compared to other cities, as coffee is rich in antioxidants. Carvalho et al.^14^ found that coffee was the food contributing most to DTAC in pregnant women. Despite the benefits of the high antioxidant content in coffee, it is important to highlight that coffee consumption during pregnancy should be moderate. Excessive caffeine intake during pregnancy is associated with low birth weight^27^, spontaneous abortion, fetal growth restriction, and an increased risk for cognitive development deficiencies, overweight, and obesity^28^. This is due to the solubility of caffeine in lipids, which allows its transfer across the blood-placental barrier, coupled with a significant decrease in the metabolic rate of caffeine in mothers. Consequently, the fetus and placenta lack sufficient enzymes for its metabolism^28^. The American College of Obstetricians and Gynecologists (ACOG) recommends that pregnant women consume no more than 200 mg of caffeine daily, which is equivalent to approximately two cups of coffee^29^.

In this study, women over 19 years of age were more likely to have a DTAC above the median, with a positive gradient observed. The likelihood was even greater in pregnant women aged 35 years old or older compared to those aged 20 to 34 years. This finding aligns with studies indicating that older pregnant women tend to have a higher-quality diet and generally consume fewer foods with high sodium, fat, and sugar content^14,30-32^. The study by Shin et al.^30^ demonstrated that pregnant women with greater adherence to a “healthy” dietary pattern, comprising cheese, coffee, dairy products, vegetables, fruits, nuts and seeds, oils, poultry, seafood, tomatoes, and a low intake of high-energy beverages, were more likely to be older.

The higher dietary quality observed in older women may be attributed to their greater discipline regarding food choices^33^. Additionally, older women are likely to have more financial stability and a more balanced life, which could grant them better access to healthier food options compared to adolescents^34^.

Pregnant women with higher levels of education were less likely to have an antioxidant intake above the median DTAC. This could be linked to the higher coffee consumption observed among pregnant women with lower education levels, supporting the findings of Zuccolotto et al.^35^, who demonstrated that women with lower educational attainment had greater adherence to a “coffee” dietary pattern, which included coffee, sugar, margarine, and butter.

Pregnant women in the second trimester were more likely to have an antioxidant intake above the median DTAC. This finding may be attributed to the fact that food consumption during pregnancy often varies by gestational trimester. In the first trimester, many women experience nausea, vomiting, and morning sickness, which can make it challenging to meet nutritional needs. In the second trimester, food consumption generally returns to normal, while in the third trimester, feelings of satiety with smaller food portions, concerns about weight gain, and heartburn can result in decreased food intake^36-38^.

The median DTAC of the pregnant women evaluated was 5.32 mmol/day. Studies assessing DTAC in pregnant women, particularly using the same method as the present study (FRAP), are limited, which complicates comparisons. Sartorelli et al.^4^ reported a median DTAC of 4.3 mmol/day in pregnant women from Ribeirão Preto (SP), a value lower than that observed in this study. The difference in DTAC values may be attributed to the inclusion of participants from multiple cities across various regions of Brazil in the present study.

A study conducted among pregnant women in Isfahan, Iran, reported a mean DTAC (12.8 mmol/day), which is twice as high as the mean observed in the present study (5.3 mmol/day)^39^. This disparity in DTAC values may be attributed to the dietary habits of the Iranian population, characterized by the consumption of antioxidant-rich foods. According to Karizaki^40^, Iranian cuisine is diverse and nutritious, with rice serving as a dietary staple. Additionally, the consumption of saffron, dates, peppers, and other spices is widespread across much of the country^40^. Similarly, a longitudinal study conducted in Bialystok, Poland, reported a mean DTAC of 12.4 mmol/day^41^. Another study carried out in Rotterdam, the Netherlands, aimed at assessing the association between DTAC and breast cancer risk in adults, found a median DTAC of 18 mmol/day^42^. These findings highlight that antioxidant intake in Brazil is lower compared to populations in high-income countries, such as Poland and the Netherlands.

The low consumption of antioxidants during pregnancy may contribute to adverse outcomes such as pre-eclampsia, gestational diabetes, and premature birth. This underscores the importance of a nutrient-rich diet with antioxidant properties to mitigate the effects of oxidative stress and prevent maternal and fetal diseases and disorders^4,5^.

This study has certain limitations. The inclusion of only public health system users in the sample necessitates caution when generalizing the findings to pregnant women in different healthcare access contexts or socioeconomic conditions. Additionally, the determination of DTAC for most foods relied on international tables, where the values may differ from those of foods produced in Brazil due to variations in genetic characteristics, soil composition, agricultural practices, climate, and cultural factors. Although priority was given to the national table by Rufino et al.^26^, the antioxidant content of most foods consumed by the study sample was characterized using international data.

Among the strengths of this study is the inclusion of data from multiple cities across the five regions of Brazil, despite the sample not being representative. This makes it the first study to assess the DTAC of pregnant women across different regions of Brazil, aiming to compare various socioeconomic factors related to dietary antioxidant consumption and to identify factors associated with this intake.

In conclusion, older pregnant women in their second trimester were more likely to have DTAC levels above the median. Conversely, women with higher levels of education consumed fewer antioxidant-rich foods. While differences in antioxidant consumption were observed among cities within the country, no significant variation was identified across the different regions of Brazil.

This study contributes to characterizing the profile of pregnant women regarding antioxidant consumption in cities across the five regions of Brazil. The low intake of antioxidants among Brazilian pregnant women raises concerns, particularly due to the potential negative impacts on maternal and child health. Although coffee was identified as the primary contributor to antioxidant consumption, it is important to highlight that increased or excessive consumption of coffee is not advisable during pregnancy due to the potential adverse effects of caffeine on fetal health.

Finally, health professionals are encouraged to emphasize the importance of a diet rich in antioxidants to pregnant women during prenatal care.
