# Supplementary material for: Factors associated with the dietary total antioxidant capacity of pregnant Brazilian women
Source: Rev Bras Epidemiol. 2025 Feb 10;28:e250002. doi: 10.1590/1980-549720250002 (PMC11809266; doi:10.1590/1980-549720250002)
Supplement: Translation

# Translation: Fatores associados à capacidade antioxidante total da dieta de gestantes brasileiras

**DOI:** 10.1590/1980-549720250002.2

**Authors:** Roberta Rejane Santos de Carvalho, Poliana Cristina de Almeida Fonseca Viola, Sandra Patrícia Crispim, Ana Karina Teixeira da Cunha França, Anderson Marliere Navarro, Bruno Feres de Souza, Franciane Rocha de Faria, Naiara Sperandio, Nathalia Pizato, Mariana de Souza Macedo, Renata Junqueira Pereira, Sylvia do Carmo Castro Franceschini, Carolina Abreu de Carvalho, Estudo Multicêntrico de Deficiência de Iodo - EMDI Brasil, Anderson Marliere Navarro, Carolina Abreu de Carvalho, Danielle Góes da Silva, Franciane Rocha de Faria, Naiara Sperandio, Jorge Gustavo Velásquez Meléndez, Míriam do Carmo Rodrigues Barbosa, Nathalia Pizato, Mariana de Souza Macedo, Renata Junqueira Pereira, Sandra Patrícia Crispim, Silvia Eloiza Priore, Sylvia do Carmo Castro Franceschini

**Affiliations:** I Universidade Federal do Maranhão, Programa de Pós-Graduação em Saúde Coletiva - São Luís (MA), Brasil.; II Universidade Federal do Piauí, Departamento de Nutrição, Campus Universitário Ministro Petrônio Portella - Teresina (PI), Brasil.; III Universidade Federal do Paraná, Programa de Pós-Graduação em Alimentação e Nutrição - Curitiba (PR), Brasil.; IV Universidade de São Paulo, Faculdade de Medicina de Ribeirão Preto, Departamento de Ciências da Saúde - Ribeirão Preto (SP), Brasil.; V Universidade Federal de Rondonópolis, Faculdade de Ciências da Saúde - Rondonópolis (MT), Brasil.; VI Universidade Federal do Rio de Janeiro - Macaé (RJ), Brasil.; VII Universidade Federal de Brasília, Departamento de Nutrição - Brasília (DF), Brasil.; VIII Universidade Federal dos Vales do Jequitinhonha e Mucuri, Programa de Pós-Graduação em Ciências da Nutrição - Diamantina (MG), Brasil.; IX Universidade Federal do Tocantins, Ambulatório de Nutrição - Palmas (TO), Brasil.; X Universidade Federal de Viçosa, Programa de Pós-Graduação em Ciência da Nutrição - Viçosa (MG), Brasil.; i Universidade de São Paulo – Ribeirão Preto (SP), Brasil; ii Universidade Federal do Maranhão – São Luís (MA), Brasil; iii Universidade Federal de Sergipe – Aracaju (SE), Brasil; iv Universidade Federal de Rondonópolis – Rondonópolis (MT), Brasil; v Universidade Federal do Rio de Janeiro – Macaé (RJ), Brasil.; vi Universidade Federal de Minas Gerais – Belo Horizonte (MG), Brasil; vii Universidade Federal do Espírito Santo – Vitória (ES), Brasil; viii Universidade de Brasília – Brasília (DF), Brasil; ix Universidade Federal dos Vales do Jequitinhonha e Mucuri – Diamantina (MG), Brasil; x Universidade Federal do Tocantins – Palmas (TO), Brasil; xi Universidade Federal do Paraná – Curitiba (PR), Brasil.; xii Universidade Federal de Viçosa – Viçosa (MG), Brasil; xiii Universidade Federal de Viçosa – Viçosa (MG), Brasil

**Keywords:** Antioxidantes, Gestação, Estresse oxidativo, Consumo alimentar, Fatores sociodemográficos

## Abstract

**Objetivo:**

Investigar os fatores sociodemográficos, maternos e gestacionais associados à capacidade antioxidante total da dieta em gestantes brasileiras.

**Métodos:**

Estudo transversal com 2.232 gestantes com 18 anos ou mais, no primeiro, segundo e terceiro trimestres de gestação de onze cidades das cinco regiões brasileiras. Foi aplicado um questionário semiestruturado para avaliação dos dados socioeconômicos, demográficos e de saúde, e o recordatório de 24h (R24h) para avaliação do consumo alimentar e análise da Capacidade Antioxidante Total da Dieta (CATd), estimada usando o método Ferric Reducing Antioxidant Power (FRAP).

**Resultados:**

A mediana de CATd foi de 5,32 mmol/dia. Aracaju (SE) apresentou a maior mediana de CATd (6,44 mmol/dia) e Palmas (TO) a menor (4,71 mmol/dia). As gestantes de 20 a 34 anos (OR 1,86; IC95% 1,26-2,76), 35 anos ou mais (OR 3,68; IC95% 2,21-6,14) e que estavam no segundo trimestre de gestação (OR 1,50; IC95%; 1,11-2,01) tiveram mais chances de estarem acima da mediana de CATd, enquanto as gestantes com maior escolaridade tinham 67% menos chance de estarem acima da mediana de CATd (OR 0,67; IC95% 0,48-0,92).

**Conclusão:**

O estudo demonstrou que há diferenças no consumo de antioxidantes em diferentes cidades do Brasil e que fatores associados, como idade, escolaridade e trimestre gestacional podem impactar na ingestão de alimentos ricos em antioxidantes. O perfil encontrado chama atenção para a importância de uma alimentação adequada e rica em antioxidantes durante o pré-natal.

## INTRODUÇÃO

A nutrição materna é um dos principais determinantes para o adequado desenvolvimento e crescimento do feto, uma vez que, nessa fase da vida, ocorre aumento das necessidades energéticas e nutricionais^1,2^.

Essas necessidades ocorrem devido ao aumento da taxa metabólica basal, à elevação do débito cardíaco, ao consumo de oxigênio e à formação de radicais livres, caracterizando a gestação como um estado de elevado estresse oxidativo^3^. Por isso, a alimentação adequada e rica em nutrientes com propriedades antioxidantes, proveniente de frutas, legumes e verduras durante a gestação é recomendada e pode prevenir distúrbios maternos e fetais, como pré-eclâmpsia, diabetes, parto prematuro, abortos espontâneos, restrição de crescimento intrauterino, entre outros^4-7^.

Há evidências de que no Brasil o consumo alimentar das gestantes é deficiente, caracterizado pelo baixo consumo de frutas e hortaliças^8^, ingestão excessiva de alimentos muito calóricos e baixo teor de nutrientes essenciais^9,10^.

A Capacidade Antioxidante Total da Dieta (CATd) é uma forma de avaliação do consumo alimentar e um marcador de qualidade da dieta que considera todos os antioxidantes presentes na alimentação do indivíduo e os efeitos sinérgicos entre eles^11-13^. Estudos que relacionam a CATd em gestantes são escassos no Brasil e no mundo e não existe uma recomendação específica de antioxidantes na gestação.

Até o momento foram encontrados dois estudos que avaliaram a CATd em gestantes, ambos com a mesma amostra, realizado em Ribeirão Preto (SP), Brasil^4,14^. Sartorelli et al.^4^ identificaram que gestantes com maior CATd tiveram menor chance de ter parto prematuro e recém-nascido de baixo peso ao nascer.

O consumo alimentar é influenciado pelas diferenças regionais e socioeconômicas, e o Brasil, devido à sua grande extensão territorial, possui grande disparidade social. Isso impacta no acesso à alimentação de qualidade e nas escolhas alimentares^15^. Embora não haja estudos que tenham avaliado a CATd em diferentes regiões do país, estudos que avaliaram outros aspectos do consumo alimentar encontraram diferenças regionais^16,17^. Pereira et al.^16^ analisaram o Índice Inflamatório da Dieta (IID) da população brasileira e verificaram que moradores da região Nordeste e Sul apresentaram dieta mais pró-inflamatória, já os da Centro-Oeste foram os que apresentaram menor IID. Portanto, é possível que, ao analisar a CATd, também sejam identificadas as diferenças regionais, o que contribuirá para o direcionamento de políticas públicas mais focalizadas em regiões de maior risco. Portanto, este estudo objetiva investigar os fatores sociodemográficos, maternos e gestacionais associados à capacidade antioxidante total da dieta em gestantes brasileiras.

## MÉTODOS

Estudo transversal com dados do Estudo Multicêntrico de Deficiência de Iodo (EMDI), desenvolvido em cidades das cinco regiões do Brasil: Viçosa (MG), Belo Horizonte (MG), Vitória (ES), Macaé (RJ), Ribeirão Preto (SP), Pinhais (PR), Brasília (DF), Rondonópolis (MT), São Luís (MA), Aracaju (SE) e Palmas (TO). Foram avaliadas gestantes em todos os trimestres, maiores de 18 anos, de risco habitual e que realizaram pré-natal no Sistema Único de Saúde (SUS). As gestantes com histórico de doença e/ou cirurgia tireoidiana ou diagnóstico de hipotireoidismo não foram incluídas no estudo.

A amostra do EMDI foi determinada a partir de uma proporção mínima estimável de 8%, com erro fixado em 50% (intervalo de 4 a 12%) e precisão de confiança de 95%, o que resultou em uma amostra aleatória simples de 177 gestantes. Foi incluído no cálculo o efeito do plano amostral (*design effect*) de 1,5, já que a amostra é complexa e foi selecionada a partir das Unidades Básica de Saúde (UBS) de cada município estudado. O tamanho da amostra aumentou para um mínimo de 266 gestantes em cada centro de coleta.

A seleção das participantes foi realizada em dois estágios: no primeiro foram sorteadas as UBS em cada cidade e no segundo estágio selecionadas as gestantes com base na lista de gestantes acompanhadas mensalmente durante o período de vigência da pesquisa, a qual constitui a base de sorteio e recrutamento do estudo. Eventualmente, em algumas unidades não foi possível obter a listagem de gestantes e estas selecionadas por sorteio a partir da ordem da lista de gestante agendadas para consulta de rotina de pré-natal, segundo a demanda de atendimento no dia em que a equipe de pesquisa se encontrava na UBS.

Os dados foram coletados no período de janeiro de 2019 a março de 2021 em UBS das onze cidades por entrevistadores previamente treinados. As gestantes foram entrevistas enquanto aguardavam pela consulta de rotina do pré-natal ou após ela. Em situações em que a gestante não tinha tempo para realizar a entrevista na própria UBS, foi feito o agendamento da entrevista em seu domicílio.

Um questionário aplicado por meio de tablets, com auxílio do aplicativo RedCap^®^, foi usado para investigar dados socioeconômicos, demográficos e de saúde, tais como regiões (Norte, Nordeste, Centro-oeste, Sudeste e Sul), faixa etária (18 a 19 anos, 20 a 34 anos, 35 ou mais), renda familiar em reais (categorizada em tercil), cor da pele (branca e não branca), vive com o companheiro (não; não, mas já viveu; sim), escolaridade (0 a 8 anos, 9 a 11 anos, 12 ou mais), chefe domiciliar (companheiro(a), ela mesma e outros), local de residência (rural e urbano), trimestre gestacional (primeiro, segundo e terceiro), IMC pré-gestacional em kg/m^2^ (baixo peso, eutrofia e excesso de peso) e IMC gestacional (baixo peso, adequado, excesso de peso). As informações de peso e altura usadas para calcular o IMC pré-gestacional foram autorreferidas pela gestante, além de ter sido feita a conferência no cartão da gestante, quando disponível. Havendo divergência entre as informações autorreferidas e as do cartão da gestante, priorizou-se os dados deste último, pois foram assinalados pelo profissional de saúde.

Os pontos de corte utilizados para classificar o estado nutricional pré-gestacional foram da Organização Mundial de Saúde (OMS): <18,5 kg/m^2^ (baixo peso), 18,5 a 29,9 kg/m^2^ (eutrofia), 25,0 a 29,9 (sobrepeso) e ≥30 kg/m^2^ (obesidade)^18^. No estudo, a categoria excesso de peso engloba as mulheres com sobrepeso e obesidade.

Para identificar o estado nutricional gestacional, o IMC foi calculado com o peso atual da gestante e classificado na curva de IMC para idade gestacional^19^.

A estimativa do consumo alimentar das gestantes foi realizada através do Recordatório de 24 horas (R24h) e foi aplicado o “método do múltiplo passo”^20^. O R24h possuía informações sobre o horário de cada refeição, se o alimento consumido era caseiro ou industrializado, a marca, o tipo ou sabor, modo de preparo, as receitas de cada preparação e, por fim, as quantidades consumidas.

Para quantificar as porções de cada alimento ou preparação foi utilizado o Manual Fotográfico de Quantificação Alimentar, com 96 fotos de porções de alimentos, preparações típicas brasileiras e formas de quantificação alimentar^21^.

Os R24h de todos os centros de pesquisa foram enviados ao Grupo de Pesquisa em Exposição Alimentar (GUPEA) na Universidade Federal do Paraná e, em seguida, digitados e analisados no software GloboDiet. A identificação do componente nutricional foi realizada tanto na Tabela Brasileira de Composição de Alimentos (TBCA) quanto na Tabela de Composição de Iodo em Alimentos (TCIA)^22,23^.

A CAT de cada alimento foi determinada multiplicando-se a quantidade de alimento ou bebida relatada no R24h pelo Ferric Reducing-Antioxidant Power (FRAP) do alimento ou bebida, e, no final, o valor obtido foi dividido por 100. Após o cálculo da CAT de cada alimento e bebida, foram somados todos os valores de CAT de todos os alimentos consumidos por cada gestante no dia, para obter o valor da CATd.

A CATd foi estimada com base em bancos de dados desenvolvidos por Carlsen et al.^24^ e Halvorsen et al.^25^. Além disso, foi utilizada uma tabela brasileira desenvolvida por Rufino et al.^26^, voltada para a análise de antioxidantes de dezoito frutas nativas, não tradicionais e frescas. Esses bancos de dados de alimentos descrevem a CAT de alimentos e bebidas e utilizam o método do poder antioxidante redutor férrico ou FRAP. Os valores de FRAP de frutas foram obtidos prioritariamente por meio da tabela brasileira^26^. Nos casos que tiveram mais de um valor de FRAP para o mesmo alimento nas tabelas, foi calculado o valor médio de FRAP. Quando um alimento não foi contemplado nas tabelas, foi considerado o valor de FRAP de um alimento do mesmo grupo botânico ou aqueles que tivessem propriedades nutricionais semelhantes.

Foi realizada a análise descritiva das variáveis sociodemográficas, trimestre gestacional e estado nutricional pré-gestacional e gestacional. As variáveis categóricas foram apresentadas por meio de frequências absolutas e relativas, e as contínuas por meio de medidas de tendência central e dispersão. Para avaliar a normalidade da distribuição das variáveis foram analisados histogramas e gráficos boxplot e por meio do teste de Shapiro-Wilk.

Os valores obtidos da CATd foram ajustados por energia e categorizados em tercis para fins de análise, tendo em vista que ainda não há um ponto de corte para a classificação de CATd. Os nutrientes consumidos pelas gestantes foram ajustados por energia usando o método residual. A quantidade de nutrientes em cada tercil de CATd foi comparada por meio dos testes de Kruskal-Wallis. Para investigar as diferenças sociodemográficas entre os tercis de CATd foram realizados o teste qui-quadrado para as variáveis categóricas e o teste de Kruskal-Wallis para variáveis contínuas.

A comparação entre as medianas de consumo de café segundo os grupos de escolaridade e a comparação das medianas de CATd entre as regiões e entre as cidades foram realizadas pelo teste de Kruskal-Wallis, e o post-hoc de Dunn para verificar a diferença entre os grupos de escolaridade, regiões e cidades.

A análise dos fatores associados à CATd acima da mediana foi estimada por Odds Ratio (OR), calculada em modelos bivariados e multivariados de regressão logística. As medidas de OR a partir da regressão logística avaliaram os fatores associados à CATd acima da mediana, conforme mostrado na Tabela 1. As variáveis explicativas incluídas no modelo multivariado foram aquelas que apresentaram p-valor <0,10 na análise bivariada, a saber: idade, escolaridade, vive com companheiro, trimestre gestacional e IMC pré-gestacional. Para análise dos dados foi utilizado o software RStudio^®^, versão 4.2.1. Em todas as análises, com exceção dos modelos bivariados, o nível de significância foi fixado em 5%.

**Tabela 1:** Fatores associados à Capacidade Antioxidante Total da Dieta acima da mediana (>5,32 mmol/dia) de gestantes participantes do Estudo Multicêntrico de Deficiência de Iodo, segundo as regiões do Brasil. Brasil, 2019.

		Capacidade antioxidante total da dieta*					
		Modelo não ajustado			Modelo ajustado†		
		OR	IC95%	p-valor	OR	IC95%	p-valor
Regiões							
	Centro-oeste	1	–				
	Norte	0,74	0,40-1,36	0,343			
	Nordeste	1,17	0,84-1,62	0,337			
	Sudeste	1,25	0,92-1,68	0,143			
	Sul	1,02	0,69-1,50	0,903			
	Renda *per capita*	1,00	0,99-1,00	0,218			
Idade (anos)							
	18 a 19	1	–		1	–	
	20 a 34	1,50	1,05-2,15	**0,024**	1,86	1,26-2,76	**0,001**
	35 ou mais	2,75	1,75-4,33	**<0,001**	3,68	2,21-6,14	**<0,001**
Cor da pele							
	Branca	1	–				
	Não branca	1,02	0,80-1,31	0,825			
Vive com companheiro							
	Não	1	–		1	–	
	Não, mas viveu	1,58	0,98-2,54	**0,056**	1,28	0,77-2,12	0,325
	Sim	1,01	0,74-1,39	0,921	0,85	0,61-1,18	0,342
Escolaridade (anos)							
	0 a 8	1	–		1	–	
	9 a 11	0,78	0,55-1,10	0,163	0,79	0,54-1,15	0,226
	12 ou	0,69	0,51-0,93	**0,015**	0,67	0,48-0,92	**0,014**
Chefe domiciliar							
	Companheiro (a)	1	–				
	Ela mesma	1,20	0,93-1,54	0,152			
	Outros	0,96	0,74-1,25	0,808			
Local da residência							
	Rural	1	–				
	Urbano	1,34	0,86-2,08	0,192			
Trimestre gestacional							
	Primeiro	1	–		1	–	
	Segundo	1,51	1,15-1,98	**0,003**	1,50	1,11-2,01	**0,006**
	Terceiro	1,30	0,99-1,71	0,050	1,28	0,96-1,71	0,087
IMC pré-gestacional							
	Eutrófico	1	–		1	–	
	Baixo peso	0,60	0,37-0,99	**0,045**	0,68	0,41-1,13	0,144
	Excesso de peso	0,92	0,74-1,15	0,515	0,86	0,69-1,09	0,231
IMC atual gestacional							
	Eutrófico	1	–				
	Baixo peso	0,77	0,54-1,09	0,146			
	Excesso de peso	0,91	0,71-1,16	0,450			

* CATd ajustado por energia usando o método residual. Mediana CATd 5,32 mmol/dia;

† Modelo de regressão logística ajustado por idade (0-19/20-34/35 ou mais), vive com companheiro (não; não, mas viveu; sim), escolaridade (0-8 anos/9-11 anos/12 ou mais anos), trimestre gestacional (primeiro, segundo e terceiro) e índice de massa corporal pré-gestacional (kg/m^2^) (baixo peso, eutrófico e excesso de peso).

CATd: Capacidade Antioxidante Total da Dieta; OR: *odds ratio*; IC: intervalo de confiança; IMC: índice de massa corpórea.

Em negrito valores com p<0,05.

Este projeto foi aprovado pelo Comitê de Ética em Pesquisa com Seres Humanos da Universidade Federal de Viçosa, universidade coordenadora do estudo multicêntrico (CAAE: 80172617.0.1001.5153). Todos os centros também submeteram o projeto ao CEP local e obtiveram aprovação.

## RESULTADOS

Foram entrevistadas 2.376 gestantes no EMDI, entretanto, 2.247 responderam ao R24h. Destas, 15 foram excluídas por falta de informações sociodemográficas, resultando em 2.232 incluídas no presente estudo (taxa de resposta=93,9%). Destas, 43% residiam no Sudeste, a maior parte (78%) tinha 20-34 anos, possuíam mediana de renda per capita de R$ 600,00, 76,6% se autodeclaravam com uma cor não branca, 78,8% viviam com o companheiro e 62,2% frequentaram por 12 anos ou mais a escola. Quanto às características da gestação, 39,8% estavam no terceiro trimestre gestacional e 47,2% estavam com excesso de peso (Tabela 2).

**Tabela 2:** Características socioeconômicas e de saúde por tercil de capacidade antioxidante total da dieta (CATd) ajustada por energia de gestantes participantes do Estudo Multicêntrico de Deficiência de Iodo (EMDI Brasil), segundo as regiões do Brasil. Brasil, 2019.

		Total	T1 (menor; n=744)	T2 (n=744)	T3 (maior; n=744)	p-valor
		n (%)	n (%)	n (%)	n (%)	
Regiões						
	Norte	89 (4,0)	32 (36,0)	36 (40,4)	21 (23,6)	0.154*
	Nordeste	553 (24,8)	189 (34,2)	169 (30,6)	195 (35,3)	
	Centro-oeste	357 (16,0)	125 (35,0)	122 (34,2)	110 (30,8)	
	Sudeste	960 (43,0)	297 (30,9)	336 (35,0)	327 (34,1)	
	Sul	273 (12,2)	101 (37,0)	81 (29,7)	91 (33,3)	
Faixa etária (anos)						
	18 a 19	219 (9,8)	86 (39,3)	78 (35,6)	55 (25,1)	**0.001** *
	20 a 34	1742 (78,0)	590 (33,9)	583 (33,5)	569 (32,7)	
	35 ou mais	271 (12,2)	68 (25,1)	83 (30,6)	120 (44,3)	
Renda familiar (tercis)						
	Mediana	600	566,66	570,83	600	0.805†
Cor de pele						
	Branca	491 (23,4)	173 (35,2)	150 (30,5)	169 (34,4)	0.386*
	Não branca	1607 (76,6)	532 (33,1)	544 (33,9)	531(33,0)	
Vive com o companheiro						
	Não	277 (13,3)	92 (33,3)	98 (35,4)	87 (31,4)	0.159*
	Não, mas já viveu	165 (7,9)	46 (27,9)	50 (30,3)	69 (41,8)	
	Sim	1.645 (78,8)	561 (34,1)	545 (33,1)	539 (32,8)	
Escolaridade (anos)						
	0 a 8	344 (16,5)	95 (27,6)	124 (36,0)	125 (36,3)	0.103*
	9 a 11	445 (21,3)	150 (33,7)	141 (31,7)	154 (34,6)	
	12 ou mais	1.299 (62,2)	458 (35,3)	424 (32,6)	417 (32,1)	
Chefe domiciliar						
	Companheiro(a)	1020 (48,6)	353 (34,6)	332 (32,5)	335 (32,8)	0.194*
	Ela mesma	560 (26,7)	180 (32,1)	175 (31,3)	205 (36,6)	
	Outros	518 (24,7)	172 (33,2)	188 (36,3)	158 (30,5)	
Local da residência						
	Rural	121 (5,8)	48 (39,7)	36 (29,8)	37 (30,6)	0.275*
	Urbano	1975 (94,2)	644 (32,6)	665 (33,7)	666 (33,7)	
Trimestre gestacional						
	Primeiro	505 (22,7)	189 (37,4)	171 (33,9)	145 (28,7)	0.053*
	Segundo	836 (37,5)	255 (30,5)	285 (34,1)	296 (35,4)	
	Terceiro	887 (39,8)	299 (33,7)	288 (32,5)	300 (33,8)	
IMC pré-gestacional (kg/m^2^)						
	Baixo peso	113 (5,6)	47 (41,6)	36 (31,9)	30 (26,5)	0.112*
	Eutrofia	946 (47,2)	300 (31,7)	332 (35,1)	314 (33,2)	
	Excesso de peso	946 (47,2)	330 (34,9)	295 (31,2)	321 (33,9)	
IMC gestacional						
	Baixo peso	276 (15,2)	94 (34,1)	104 (37,7)	78 (28,3)	0.235*
	Eutrófico	683 (37,6)	223 (32,7)	220 (32,2)	240 (35,1)	
	Excesso de peso	859 (47,2)	296 (34,5)	273 (31,8)	290 (33,8)	

* Pearson's Chi-squared test;

† Kruskal-Wallis rank sum test.

Em negrito valores com p<0,05.

A mediana de CATd foi de 5,32 mmol/dia. A região Sudeste foi a que obteve maior mediana de CATd (5,48 mmol/dia) e a Norte foi a com menor mediana (4,71 mmol/dia) (Figura 1). Aracaju (SE) foi a cidade com maior mediana de CATd, (6,44 mmol/dia) e Palmas (TO) a com menor mediana (4,71 mmol/dia).

Houve diferença nas medianas de CATd entre as cidades (p<0,001), sendo que a mediana de CATd em Palmas foi menor que a mediana de CATd em Aracaju (SE) e menor que a mediana de CATd em Belo Horizonte (MG). Não houve diferença entre as medianas de CATd nas regiões (Figura 1).

**Figura 1:** Mediana de capacidade antioxidante total da dieta ajustada de gestantes do Estudo Multicêntrico de Deficiência de Iodo. A) Mediana de capacidade antioxidante total da dieta em mmol/dia ajustada por energia de gestantes, segundo a região do Brasil e B) Mediana de capacidade antioxidante total da dieta em mmol/dia ajustada por energia de gestantes, segundo a cidade. Brasil, 2019.

CATd: Capacidade Antioxidante Total da Dieta.

As gestantes com 35 anos ou mais estavam em maior proporção no maior tercil de CATd (p=0,001), conforme Tabela 2. Gestantes que apresentaram maior consumo de carboidrato, fibras, ferro, magnésio, potássio, cobre, retinol, vitamina D, vitamina E, tiamina, vitamina B2, vitamina B6, folato e iodo também estavam no maior tercil de CATd (Tabela 3).

**Tabela 3:** Características da dieta de acordo com os tercis da capacidade antioxidante total estimada da dieta ajustados por energia de gestantes participantes do Estudo Multicêntrico de Deficiência de Iodo, segundo as regiões do Brasil. Brasil, 2019.

Tercis CATh ajustada				
Nutrientes	T1 (Menor)	T2	T3 (Maior)	p-valor*
Carboidrato (g)	230,46	240,25	251,67	**<0,001**
Proteína (g)	77,55	73,64	72,78	**0,001**
Lipídios (g)	68,41	65,83	62,65	**<0,001**
Ácidos graxos saturados (g)	23,02	21,30	20,19	**<0,001**
Ácidos graxos monoinsaturados (g)	20,81	19,59	18,64	**<0,001**
Ácidos graxos poli-insaturados (g)	15,54	15,74	15,32	0,269
Ácidos graxos trans (g)	1,52	1,44	1,37	**0,001**
Colesterol (mg)	266,98	241,74	234,10	**<0,001**
Fibras (g)	14,36	18,58	20,68	**<0,001**
Cálcio (mg)	448,06	467,28	461,26	0,435
Ferro (mg)	10,16	10,70	11,16	**<0,001**
Sódio (mg)	2326,23	2293,26	2252,85	0,214
Magnésio (mg)	211,47	239,85	262,85	**<0,001**
Fósforo (mg)	992,18	1003,61	1010,64	0,336
Potássio (mg)	1855,81	2133,86	2418,02	**<0,001**
Manganês (mg)	3,40	4,08	3,84	**<0,001**
Zinco (mg)	10,52	10,49	10,11	0,572
Cobre (mg)	1,04	1,18	1,23	**<0,001**
Selênio (mcg)	36,51	36,58	35,90	0,328
Vitamina A (mcg)	333,99	372,16	406,15	**<0,001**
Vitamina D (mcg)	2,02	2,26	2,76	**<0,001**
Vitamina E (mcg)	5,25	5,68	5,89	**<0,001**
Tiamina (mg)	0,91	0,93	1,03	**<0,001**
B2 (mg)	0,99	1,15	1,31	**<0,001**
B3 (mg)	14,83	14,23	14,33	0,387
B6 (mg)	0,59	0,67	0,71	**<0,001**
B12 (mcg)	4,00	3,62	3,20	**<0,001**
Vitamina C (mg)	57,49	72,36	71,28	**<0,001**
Folato (eq)	293,10	351,56	409,17	**<0,001**
Iodo (mcg)	115,83	121,36	124,15	**0,002**

* *Kruskal-Wallis rank sum test*.

CATd: Capacidade Antioxidante Total da Dieta. Em negrito valores com p<0,05.

Os cinco alimentos com maiores médias de contribuição de CATd por centro estão detalhados na Tabela Suplementar 1 e os alimentos com maiores medianas de contribuição de CATd por categorias de escolaridade e por região estão detalhados na Tabela 4.

**Tabela 4:** Alimentos com maiores medianas de contribuição de Capacidade Antioxidante Total da Dieta em gestantes participantes do Estudo Multicêntrico de Deficiência de Iodo, segundo a escolaridade e a região. Brasil, 2019.

Escolaridade		Mediana de consumo diário (mmol/dia)
0 a 8 anos		
	Café	1,86
	Feijão	0,93
	Arroz	0,14
	Cebola branca	0,02
	Louro, folhas	0,00
9 a 11 anos		
	Feijão	0,97
	Café	0,93
	Arroz	0,14
	Cebola branca	0,02
	Louro, folhas	0,00
12 anos ou mais		
	Café	1,04
	Feijão	0,85
	Arroz	0,13
	Cebola branca	0,01
	Louro, folhas	0,00
Região		Mediana de consumo diário (mmol/dia)*
	Feijão	0,97
	Café	0,52
	Arroz	0,14
Nordeste		
	Café	1,8
	Feijão	0,42
	Arroz	0,13
Sudeste		
	Feijão	0,97
	Café	0,76
	Arroz	0,14
Centro-oeste		
	Feijão	0,97
	Café	0,78
	Arroz	0,14
Sul		
	Café	0,76
	Feijão	0,39
	Arroz	0,13

* foram inseridas as medianas dos três alimentos que mais contribuíram para a Capacidade Antioxidante Total da Dieta por região, pois os valores das medianas dos 4^o^ e 5^o^ alimentos foram muito pequenas, aparecendo como zero no arredondamento.

Conforme a Tabela Suplementar 1, a média de contribuição de café para a CATd em Aracaju foi quase o dobro, comparado à alguns centros.

Houve diferença na mediana de consumo de café entre os grupos de escolaridade (p=0,009). A mediana de consumo de café das gestantes com 0 a 8 anos de estudo foi maior que as medianas de consumo de café de gestantes com escolaridade de 9 a 11 anos e de 12 anos ou mais.

Em um gradiente positivo, as gestantes adultas apresentaram maior chance de ter uma ingestão de antioxidantes acima da mediana de CATd, sendo 1,86 vezes maior para aquelas com 20 a 34 anos (OR 1,86; IC95% 1,26-2,76) e 3,68 vezes maior para as com 35 anos ou mais (OR, 68; IC95% 2,21-6,14). As gestantes com maior escolaridade (OR 0,67; IC95% 0,48-0,92) tiveram 67% menor chance de estarem acima da mediana de CATd em relação às gestantes de menor escolaridade (Tabela 1). Aquelas que estavam no segundo trimestre apresentaram 1,5 vezes mais chances de estarem acima da mediana de CATd (OR 1,5; IC95% 1,11-2,01), quando comparadas às no primeiro trimestre (Tabela 1).

## DISCUSSÃO

O presente estudo avaliou os fatores associados à CATd de gestantes brasileiras usuárias da rede pública de saúde e observou que a mediana de CATd entre as gestantes foi de 5,32 mmol/dia. Em um gradiente positivo, as gestantes que tinham 35 anos ou mais tiveram maior chance de ingerir antioxidantes acima da mediana de CATd. A gestantes que estavam no segundo trimestre tiveram maior chance de estarem acima da mediana de CATd, no entanto, observou-se que aquelas que tinham maior escolaridade tiveram menor chance de estarem acima da mediana de CATd. A região Sudeste foi a região com maior CATd.

Dentre as cidades brasileiras avaliadas no presente estudo, Aracaju (SE) foi a que obteve maior CATd (6,44 mmol/dia). Esse resultado está relacionado ao maior consumo de café em comparação às outras cidades, uma vez que esse alimento é rico em antioxidante. Carvalho et al.^14^ mostrou que o café foi o alimento que mais contribuiu para a CATd em gestantes. Apesar do benefício do maior conteúdo de antioxidantes no café, é importante salientar que durante a gestação o consumo de café deve ser moderado, pois a alta ingestão de cafeína na gravidez está associada a baixo peso ao nascer^27^, aborto espontâneo, restrição de crescimento fetal e risco aumentado para deficiências no desenvolvimento cognitivo, excesso de peso e obesidade^28^. Isso se deve à solubilidade da cafeína em lipídios, o que possibilita a sua transferência pela barreira hemato-placentária e à diminuição significativa da taxa metabólica da cafeína nas mães, logo, nem o feto, nem a placenta tem enzimas suficientes para o seu metabolismo^28^. Segundo o American College of Obstetricians and Gynecologists (ACOG), aconselha-se o consumo diário de 200 mg de cafeína por gestantes, o que equivale a duas xícaras de café^29^.

Neste estudo, mulheres com mais de 19 anos tinham mais chances de estarem acima da mediana de CATd e notou-se um gradiente positivo, pois a chance no grupo com mais de 35 anos foi ainda maior que nas gestantes com 20 a 34 anos. Esse achado está de acordo com estudos que evidenciaram que gestantes com maior idade possuem maior qualidade da dieta e tendem a consumir menos alimentos com alto teor de sódio, gordura e açúcar^14,30-32^. O estudo de Shin et al.^30^ mostrou que as gestantes com maior adesão ao padrão “saudável”, composto por queijo, café, laticínios, verduras e legumes, frutas, nozes e sementes, óleos, aves, frutos do mar, tomate e baixo consumo de bebidas de alto teor energético eram propensas a serem mais velhas.

A qualidade da dieta em mulheres mais velhas pode estar relacionada ao fato de estas serem mais disciplinadas em relação à alimentação^33^. Além disso, pode ser que essas mulheres tenham uma vida mais estável e equilibrada do ponto de vista financeiro, podendo ter acesso a uma alimentação mais saudável quando comparadas às adolescentes^34^.

As gestantes com maior escolaridade tinham menor chance de terem uma ingestão de antioxidantes acima da mediana de CATd. Isso pode estar relacionado ao maior consumo de café entre as gestantes de menor escolaridade, corroborando Zuccolotto et al.^35^, que mostraram que as gestantes de menor escolaridade tiveram maior adesão ao padrão “café”, composto por café, açúcar, margarina e manteiga.

As gestantes no segundo trimestre tinham mais chance de estarem acima da mediana de CATd. Esse achado pode ter relação com o fato de que o consumo alimentar durante a gestação tende a diferir de acordo com os trimestres gestacionais. No primeiro trimestre algumas mulheres apresentam enjoos, náuseas e vômitos, o que dificulta o suprimento das necessidades nutricionais nesse período. No segundo trimestre o consumo alimentar tende a retornar à normalidade e no terceiro trimestre a sensação de saciedade com pouco consumo de alimentos, o medo de ganhar peso e a azia podem levar à diminuição da ingestão alimentar^36-38^.

A mediana de CATd das gestantes avaliadas foi de 5,32 mmol/dia. Há poucos estudos com gestantes que tenham avaliado a CATd, especialmente utilizando o mesmo método do presente estudo (FRAP), o que dificulta a comparação desse resultado. O estudo de Sartorelli et al.^4^ com gestantes em Ribeirão Preto (SP) encontrou uma mediana de CATd de 4,3 mmol/dia, portanto, inferior à do presente estudo. A diferença dos valores de CATd pode estar relacionada ao fato de a amostra do presente estudo envolver diversas cidades, em diferentes regiões do Brasil.

Um estudo com gestantes de Isfahan, no Irã, encontrou média de CATd (12,8 mmol/dia) duas vezes maior que a do presente estudo (5,3 mmol/dia)^39^. A diferença nos valores de CATd pode ser explicada pelos hábitos alimentares da população iraniana, caracterizados pelo consumo de alimentos ricos em antioxidantes. Segundo Karizaki^40^, a culinária iraniana é muito diversificada e nutritiva, e a alimentação tem como base o arroz. Além disso, na maior parte do país o consumo de açafrão, tâmaras, pimentas e outras especiarias é muito comum^40^. Um estudo longitudinal realizado em Bialystok, na Polônia, mostrou uma CATd média de 12,4 mmol/dia^41^. Em outro estudo realizado em Roterdã, na Holanda, com o objetivo de avaliar a associação da CATd e o risco de câncer de mama em adultas, a mediana de CATd foi de 18 mmol/dia^42^. Portanto, nota-se que a ingestão de antioxidantes no Brasil é menor quando comparada à de populações de países de alta renda, como a Polônia e a Holanda.

Esse baixo consumo de antioxidantes na gestação pode resultar em pré-eclâmpsia, diabetes gestacional e parto prematuro, por exemplo. Por isso a importância da alimentação rica em nutrientes com propriedades antioxidantes como forma de proteção contra os efeitos do estresse oxidativo e prevenção de doenças e distúrbios maternos e fetais^4,5^.

O presente estudo possui algumas limitações. O fato de a amostra ter sido apenas de usuárias da rede pública de saúde implica em maior cautela ao extrapolar para outras gestantes em diferentes cenários de acesso à saúde ou condição socioeconômica. Destaca-se, ainda, que, para a determinação da CATd da maioria dos alimentos, foram utilizadas tabelas internacionais, nas quais os valores podem divergir dos alimentos produzidos no Brasil, devido a diferenças em aspectos genéticos, do solo, de métodos agrícolas, clima e cultura. Embora tenha sido dada prioridade para a tabela nacional de Rufino et al.^26^, o conteúdo antioxidante da maioria dos alimentos consumidos pela amostra do presente estudo foi caracterizado através das tabelas internacionais.

Entre os pontos fortes do estudo, destacamos que, embora a amostra não seja representativa, foi utilizada uma amostra com dados de várias cidades das cinco regiões do Brasil. Dessa forma, este é o primeiro estudo que avalia a CATd de gestantes em diferentes regiões do Brasil, buscando comparar os diversos fatores socioeconômicos relacionados ao consumo de antioxidantes da dieta e identificar fatores associados a esse consumo.

Em conclusão, as gestantes com maior idade e que estavam no segundo trimestre gestacional apresentaram maiores chances de estarem acima da mediana de CATd. Todavia, mulheres de maior escolaridade consumiam menos alimentos antioxidantes. Há diferenças no consumo de antioxidantes em diferentes cidades do país, mas não houve diferença no consumo de antioxidantes em diferentes regiões do Brasil.

O presente estudo ajuda a traçar um perfil de gestantes quanto ao consumo de antioxidantes em cidades das cinco regiões do Brasil. Preocupa o baixo consumo de antioxidantes entre as gestantes brasileiras, sobretudo considerando o impacto negativo que isso pode ter na saúde materno-infantil. Embora o café tenha sido o alimento com maior contribuição para o consumo de antioxidantes, é importante destacar que não é recomendado o aumento do consumo ou o consumo excessivo desse alimento entre gestantes, dados os potenciais malefícios da cafeína à saúde do feto.

Finalmente, encorajamos que os profissionais de saúde durante o acompanhamento pré-natal reforcem a importância de uma alimentação rica em antioxidantes às gestantes.
